# Sex and Regeneration

**DOI:** 10.3390/biology10090937

**Published:** 2021-09-19

**Authors:** Moshe Reuveni

**Affiliations:** Plant Science Institute, ARO, Volcani Institute, 68 Hamakabim Rd., P.O. Box 15159, Rishon LeZion 7528808, Israel; vhmoshe@agri.gov.il

**Keywords:** regeneration, asexual propagation, multicellular organisms, wound repair, maturation

## Abstract

**Simple Summary:**

Tissue regeneration is found in plants and some animal species. The regeneration process is ubiquitous to all multicellular organisms. Regeneration ranges from healing wounded tissue to whole body neoforming (remaking of the new body). In this larger context, regeneration is one facet of two propagation schemes that dominate the evolution of life. Multicellular organisms can propagate asexually or sexually, and regeneration is a form of asexual propagation. The hypothesis presented here claims that the ability to regenerate is determined by the sexual state of the multicellular organisms (from simple animals such as hydra and planaria to plants and complex animals). The above hypothesis is supported by showing evidence that many organisms, organs, or tissues show inhibited or diminished regeneration capacity when in reproductive status compared to organs or tissues in nonreproductive conditions or by exposure to sex hormones.

**Abstract:**

Regeneration is usually regarded as a unique plant or some animal species process. In reality, regeneration is a ubiquitous process in all multicellular organisms. It ranges from response to wounding by healing the wounded tissue to whole body neoforming (remaking of the new body). In a larger context, regeneration is one facet of two reproduction schemes that dominate the evolution of life. Multicellular organisms can propagate their genes asexually or sexually. Here I present the view that the ability to regenerate tissue or whole-body regeneration is also determined by the sexual state of the multicellular organisms (from simple animals such as hydra and planaria to plants and complex animals). The above idea is manifested here by showing evidence that many organisms, organs, or tissues show inhibited or diminished regeneration capacity when in reproductive status compared to organs or tissues in nonreproductive conditions or by exposure to sex hormones.

## 1. Introduction

In the past eras, there was a concentration of research efforts of biologists in general and plant scientists in particular to understanding the molecular basis and function of a handful of organisms. These efforts successfully explain the molecular basis and function of many biological phenomena, ranging from cell division in yeast to photosynthesis mechanism in plants to the basis of genetic diseases in humans. However, tissue regeneration in all phyla received much less attention, albeit its therapeutic and agronomic importance. Usually, regeneration was viewed as a curiosity or an agronomic practice only in plant biology, not a purely scientific process, although it has been used in agriculture for at least hundreds and maybe thousands of years. In animals, regeneration was regarded as a rare phenomenon since it was seldom observed or noticed. However, in the last decades, the understanding that tissue regeneration is a widely distributed phenomenon in the animal kingdom and that it is not necessarily connected to embryonic development can contribute to medicine and agriculture alike. Although our world is rich in organismal diversity, some fundamentally common threads are ubiquitous to all multicellular organisms, such as lipid membranes to enclose the active biochemical zones in all cells; Nucleic acids as genetic material to transfer information between generations and to store operational code for cellular and organismal function; and the ability to regenerate wounds and tissues as a survival mechanism against the harsh environment.

Regeneration is principally viewed as a unique plant and some animal species process that is performed in response to wounding, but it can be considered in a larger context. In this larger context, regeneration is one facet of a whole reproduction scheme that dominates the evolution of life and is part of the life history theory. There are two main modes of multicellular organism propagation from generation to generation. The first and most common organismal model of multiplication is sexual reproduction. During organismal sexual proliferation, two gametes (male and female) of the species must exist, meet and exchange genes. Thus, a new organism arises that contains the genetic heritage of its predecessors.

The second and probably evolutionary earlier mode of multicellular organism multiplication is the vegetative or asexual reconstruction of an entire organism, budding or emerging from an older version. Fundamentally asexual propagation can be viewed as the regeneration of a group of cells (multicellular organisms or tissues) to a whole body and not from an embryo. Parthenogenesis (or apomixes) in the context of asexual regeneration is excluded because it is part of an ordered developmental program where cells change to embryos and develop into a whole organism via the embryonic program but without fertilization. Epimorphic regeneration is one facet of asexual propagation that produces a body section such as a limb or a root. Epimorphic regeneration in animals and plants involves forming a blastema in animals and callus in plants that is necessary for proper organ regeneration to occur; in both cases, these are considered undifferentiated cells capable of regenerating neoformed tissues.

This mode of multicellular organism multiplication is more commonly known as asexual reproduction. In asexual reproduction, no wounding is necessary to induce the process. Some examples of asexual reproduction not via parthenogenesis are the budding of Hydra [1], flatworms [2], palm tree pups [3], and Kalanchoe daigremontiana [4]. The new organism outgrows from the older version and contains the exact genetic heritage of its predecessor.

Wound repair in multicellular organisms can be viewed as a particular instance of regeneration. For example, liver tissue repair [5] can be considered asexual multiplication of liver tissue according to a preplanned structural program. The process of liver repair or regeneration is exploited in medicine for transplant and artificial liver construction [5]. The ability to repair wounds varies between organisms and between organs of the same multicellular organism. The mechanisms by which cells in a particular organ or organism gain potency and subsequent ability to regenerate or repair entire organs or organisms are unknown. The capability of multicellular organisms to regenerate and repair wounds decreases as the organism ages. The age-related decline in regenerability and wound repair is universal across kingdoms [6,7,8]. Regenerative capacity has been under continual study for nearly a century and is of great interest to scientists. Multicellular organisms vary in their regeneration ability. Some tissues display high regeneration capability even in the same plant cultivar or animal genera, whereas others display no regeneration. Cells in multicellular organisms can be divided into old cells or mature cells, hence tissue and organs. Old cells or tissue are chronologically aged cells; for example, the first leaves in a plant are old; brain cells are the original cells created during development. On the other hand, the leaves around the flowers are mature; skin cells at the chronological age of 40 years are only up to 40 days old but are mature cells. Repeatedly, juvenile tissues or individuals exhibit high regenerative capacity than older tissues or individuals. Why is age-dependent regeneration so prevalent, maybe the origin of the differences in regeneration capacity is ubiquitous to all organisms?

## 2. Types of Regeneration

Several types of regeneration are distinguished in plants [9] and animals [10]. All multicellular organisms can regenerate damaged tissues. The simplest is repairing wounds either with a scar or callose tissue or returning to the original tissue. In plants, the wound repair process is widely used for in vitro propagation of plants in agriculture. Plants can be grafted on various scions, and a successful union is also a form of wound repair and thus regeneration. Another type of regeneration is organ completion, as seen in animals as a fingertip regeneration or in plants as root tip regeneration [10,11,12,13]. In this type of regeneration, the organ that was slightly amputated can repair itself. In mammals, a more complex form of organ completion regeneration is limb, fin, retina, or liver regeneration [10]. Plants do not complement organs such as leaf and root when severed above the tip. Nevertheless, plants develop callose tissue (the plant equivalent to fibrotic scar tissue), similar to what happens when a mammalian limb is amputated. Plants can undergo regeneration only if the first few millimeters of their main root are removed [12,13].

Plants and some animals can regenerate whole bodies such as *Hydra* (phylum Cnidaria) or flatworm (Platyhelminthes) that regenerate half or entire bodies when severed; plants can regenerate roots from severed stems (where roots did not exist before). When the environment of a leaf tissue segment is manipulated with phytohormones [9,13,14], or the environment of cultured mouse neonatal dermal fibroblasts is manipulated by chemicals [15], regeneration occurs to different degrees. Plants can regenerate whole shoots or roots, and animal cells can regenerate stem cells [15] or even entire animals [16]. Plant cells’ plasticity is revealed in the ability to form shoots or roots from somatic cells and the capacity to generate entier embryos without fertilization, a process is known as somatic embryogenesis, not to confuse with parthenogenesis with is the formation of an embryo in an unfertilized egg cell. It seems safe to determine that most multicellular organisms retain the ability to regenerate tissues to some extent, and regeneration is a ubiquitous trait ranging from local lesion repair to whole body neoforming.

Regeneration in all multicellular organisms is the aggregate of developmental processes responding to external and internal cues. Analysis of the different regeneration phases has proven complicated as well as determining each specific phase’s regulation. The regeneration process is usually divided into three phases, competence, induction, and development [9,17]. The mechanisms underlying the competence of regeneration or its acquisition competence remain largely elusive. The molecular-mechanistic comparison can only be made within Kingdome. The molecular-mechanistic of regeneration or development of plants is very different from that of animals. Just a simple example is that plant cells are not motile and possess an external rigid wall, while animal cells are motile and are encapsulated in a rigid cell wall.

## 3. Sex and Regeneration

Plant survival depends on the organism’s genomic flexibility; plants can not escape predation or physically move away from harsh environmental conditions. Nevertheless, plants respond to their environment by changing growth habits and phenotypes. Plants and animals differ significantly in their developmental mode and their reactions to damage and the environment. However, both plants and animals can repair wounded tissue and regenerate organs. Plant cell walls prevent cellular mobility within the plant. Thus, plants must repair or regenerate damaged tissue via the neoforming of existing somatic cells and manufacture the new tissue by cell division of the neoformed new cells. Therefore, as compensation, plants evolved a repair mechanism that allows them to survive at extreme wounding or absent reproductive partners. Plants can be reproduced from cuttings of sections of stems, roots, and bulbs, and some are able to create new embryos from somatic cells. While decades of studying plant tissues’ ability to regenerate a complete fertile shoot or root system after induction, the mechanisms by which somatic cells acquire pluripotency and neoform new entire plant organs remain unknown [14].

There are two types of proliferation modes in nature: sexual and asexual, and regeneration is a form of asexual reproduction. Plants can switch from sexual and asexual propagation, especially annual plants such as trees or bushes. Chronological age influences plant rooting ability or shoot regeneration [18,19]. Zhang et al. [6] showed that decreasing *miR156* levels, a chronologically regulated microRNA in plants, controls shoot regeneration from leaf slices of tobacco plants by steadily increasing *SQUAMOSA PROMOTER BINDING PROTEIN-LIKE* (*SPL*) mRNA levels [6]. The increase in *SPL* mRNA levels was correlated to a progressive drop in the regeneration of shoots from tobacco and *Arabidopsis* tissue [6]. *SPLs* were shown to regulate multiple age-related processes, such as embryonic pattern formation, juvenile-to-adult phase transition, the timing of flowering, and shoot regeneration ability [6,20,21,22,23,24,25,26,27]. Thus, the juvenile to adult transformation is regulated by a gene network containing at least miR156 that affects the *SPL* gene family members and downstream of *SPL* like *FLOWERING LOCUS T* (*FT*) gene the process of juvenile-to-adult phase transition [28].

Reports in past years have shown that the flowering stage reduces rooting, such as Rhododendron, *Camellia*, *Coleus*, *Vaccinium*, *Taxus,* and broccoli shoots [29,30]. Even earlier, in the 1940s of the last century, Wilton [31] showed that little or no cambial activity as a marker for stemness in plants was disappearing during the flowering stage in flowering plants [31]. While the flowering bud represses rooting, the removal of these buds increases rooting [32]. The above effects indicate that it might not be that chronological age affects regeneration, but maintaining cell division activity in plants is associated with the plants’ sexual maturity state. It is a common practice in rooting vegetative cuttings from plants to use juvenile tissue.

Rooted cuttings from trees vary between species and even individual tree clones within species, cuttings taken from trees at the juvenile stage regenerate roots more efficiently and rapidly than cuttings from trees at the adult stage, e.g., *Douglas fir* [33], radiata pine [34,35], and white spruce [36] as examples. More detailed research evaluated the root regeneration competence of cuttings throughout the year when trees go through vegetative and reproductive (flowering) cycles. For example, *Cabralea canjerana* (Vell) is a valuable crop tree that flowers between August to October. When 11 clones of *Cabralea canjerana* were examined for root regeneration of cuttings during the year, 6 out of the 11 clones exhibited reduced root regeneration during the reproductive season [37]. There was no data in the paper if the various clones flowered differently. The root regeneration ability from stem cuttings of four woody plant species from the eastern Madagascar tropical forest was tested [38]. The species tested were *Aphloia theiformis* flowering time September–November, *Ilex mitis* flowering time Sep-Dec, *Prunus africana* flowering time September–November (Flora of Zimbabwe https://www.zimbabweflora.co.zw/index.php) all the plants’ flower during the cold season in Madagascar. Root regeneration from cuttings segments of stems was dependent on the season when the cutting was obtained. Root regeneration was maximal during the hot season and very low during the flowering season, the cold season [38]. Stem cuttings of myrtle, flowering time June to October, were sampled all through the year and showed a significant time-based disparity in the ability to regenerate roots [39]. Root regeneration dropped from 70% during December to February vegetative season and reached 70%, to less than 10% during the flowering period of June to August.

Florigen, a mobile plant sexual state determining phytohormone, is encoded by the *FLOWERING LOCUS T* (*FT*) genes or *FT*-like genes in flowering plants [40,41]. *FT* gene family functions as a general growth hormone regulating shoot or root architecture and promoting organ-specific and age-related determinate growth [40]. *FT* genes show distinctly different expression patterns during development; timing, tissue specificity, and response to photoperiod are varied [42]. Controlling florigen levels after induction of flowering showed that florigen promotes lateral shoot growth independently of its effect on the phase transition from vegetative to reproductive development [43]. Florigen mutants affect lateral shoot formation differently; thus, the florigen genes are central to the floral transition and shoot development [43]. The effect of the plant sex peptide phytohormone florigen on regeneration was tested recently and was shown to reduce both shoot and root regeneration [8]. This report defines that the chronological age and sexual maturation of tissue regeneration in plants are distinct when regeneration is examined, putting plant and all multicellular organisms that show diminished regeneration capacity when reaching sexual maturity.

In animals, regeneration is primarily attributed to local or migratory stem cells activity. In plants, locating stem cells’ presence during the initial stages of regeneration has been elusive [44] or shown not to be needed for tissue regeneration [13]. Plant stem cells reside in specific rigid locations in the plant apices of the root and shoot or leaf base and buds. Plant stem cells are not capable of moving to the wound site to repair or regenerate the tissue. The stem cell niches are not present in excised leaf, root, or stem tissue and develop after the initial stages of tissue regeneration. The initial phase of regeneration, competence, is strongly affected by juvenility across kingdoms. Plant stem cells’ developmental program during regeneration, once regeneration starts, is similar to these processes and regulation of stem cell activity during post-fertilization embryogenesis. Juvenility is associated with an enhanced regenerative ability or vice versa maturity with a deceased regeneration. For example, mature plants exhibit a lower regenerative capacity when mature plants’ regenerative capacity declines [6] as plants turn to reproductive age. In mice’s adolescent state affects tissue repair, a type of regeneration [45] and mice cardiomyocytes stem cells amount and turnover decrease with age [46]; juvenile salamander can regenerate a limb faster than an adult [47]. The juvenility or maturity status of the tissue is part of the control of plants’ and animals’ regenerative capacity.

The endogenous sex hormone estrogen may enhance bone marrow mesenchymal stem cells vascular endothelial growth factor production. However, not much information exists on testosterone’s effect on stem cell function. Testosterone decreases growth factor production in stem cells, and the removal of testosterone’s deleterious effects via castration proves beneficial in growth factor production [48]. Fallopian tube epithelial cells respond to the sex steroid hormones estrogen or estradiol and progesterone by altering gene expressions. These cells show in response to the above sex hormones changes in stemness markers [49]. The neural stem cells model also indicates that cell proliferation and differentiation are affected by steroid sex hormone [50]. Another example of sex steroid hormones’ role in stem cell activity is in mice cardiac stem cells by increasing proliferation, improving telomerase activity, and reducing DNA damage [51]. Castration of male mice leads to increased longevity of stem cells [21,52]. It seems that in multicellular animal models, stem cell performance (or regeneration) decreases with age and sexual maturity that go together. However, in some multicellular organisms, chronological age is separated from sexual maturity [53], and tissue regeneration does not decrease with chronological age but is correlated to sexual maturity. If sexual maturity is eliminated (by castration) or by vegetative growth in the case of annual trees, stem cell performance or root regeneration does not decay. The concept that sexual maturity is antagonistic to stem cell functioning and thus to regeneration may be a functional determinant in plants. The ability of annual plant stem cell niches to revert from sexual to asexual state and the on/off the appearance of the plants’ sex hormone florigen may also be a contributing factor to plants’ longevity [54].

The two dominant models in regeneration research in animals are zebrafish, which regenerate caudal fins throughout life, and Axolotl, which can regenerate limbs both as juveniles and adults. Regenerative capacity decays in mammals such as humans and mice as they become chronologically older, but the organisms stop growing shortly after reaching sexual maturity, unlike Axolotl. The highly regenerative amphibian Axolotl reached sexual maturity about one year after hatching but never stopped growing [55]. It has been hypothesized that Axolotl’s exceptional regenerative capacity is the source for slowing aging-related diseases [55] and may continue its growth. However, axolotl regeneration also decreases with time as the animal ages [55] from weeks in juvenile to months in sexually mature adult animals [55,56]. Neotenous Axolotl adults’ regeneration rate is lower than larval (juvenile) animals [56]. In some metamorphic amphibians that can regenerate as tadpoles, regeneration ability diminishes when metamorphosis occurs to a sexually mature adult animal [57]. Induction of metamorphosis in Axolotl adversely affects their regeneration percentage and rate [56]. No paper shows a direct effect of sex steroid hormones on Axolotl limb regeneration; however, some indirect evidence may point to that regardless. As stated above, inducing metamorphosis in Axolotl is achieved by thyroid hormones (TH). Axolotls with TH treatment have a reduced ability to regenerate and a decreased number of cells proliferating in the limb and heart [58]. Hypothyroidism and Hyperthyroidism are associated with levels of steroid sex hormones in both female and male humans [59] and probably in other animals. Thus, suggesting that increasing TH to induce metamorphosis in Axolotl causes an increase in sex hormones and diminishes regeneration capacity.

Zebrafish heart regeneration is enhanced by estrogen supplementation and suppressed by the estrogen-antagonist tamoxifen [60]. Brain-derived neurotrophic factor is thought to be involved in telencephalic regeneration after injury in zebrafish [61]. Sex steroid hormones were shown to affect the expression and activity of Brain-derived neurotrophic factors [62]. Upon amputation of the pectoral fin in male zebrafish, the regeneration of the breeding tubercles (unique male structure) requires the presence of androgens and is inhibited by estrogen [63]. There is also a difference in regeneration ability between male and female zebrafish [60,64], indicating the involvement of sex hormones in regeneration. In both vertebrate animal models that have been extensively researched, there is sparse data on the effect of sex hormones on regeneration, albeit there are differences between juvenile and adult animals in regeneration capacity.

Planarian’s asexual propagation is by body splitting or autotomy and subsequent regeneration. Certain planarians will develop hermaphroditic sexual reproductive organs. The sexual planarians are divided into two groups asexual animals that may, under certain conditions, develop sexual reproductive organs; these are acquired sexuality worms and native sexual worms, which will reproduce only via sperm exchange [65]. Sexual planarians will also regenerate; for example, *Schmidtea mediterranea* regenerates just as well in sexual compared to asexual. However, it takes a couple of days longer (Catherine McCusker personal communication). Planarians, as model animals for regeneration studies, are used for experimentation on tissue regeneration. Many factors affect the Planarians’ regeneration capacity, among them; chemicals, temperature, and seasonal factors. Asexual planarian *Dugesia ryukyuensis* grows sexual organs and undergoes sexual propagation if fed with crushed adults of the oviparous planaria *Bdellocephala brunnea* [66]. Fukushima et al. [67] determined that androgen is present in *Bdellocephala brunnea*. The level of the steroid sex hormone varies seasonally [67]. External application of estradiol enhance the regeneration process slightly in *Girardia* *tigrina*. However, regeneration in *Girardia* *tigrina* was reduced significantly by applying testosterone and enhanced by estradiol [68]. When *Dugesia ryukyuensis* worms were fed, *Bdellocephala brunnea* crushed worms, sexualization changed according to the sexualization of the food animals [66]. In the cold season, winter, planarians developed all the sexual organs. The rate of head regeneration in *Dugesia tigrina* in the summer season exceeded the values of those in the winter, autumn, and spring season [69].

Hydrozoans breed sexually by combining gametes from male and female individual animals when the environmental condition becomes unfavorable (autumn season). Hydrozoans reproduce asexually by budding a new smaller animal during the summer season. When environmental conditions are favorable for growth, budding is the widespread reproduction method. Regeneration is not only the immediate response to injury, but it is an integrated component of the normal Hydrozoans life cycle, taking part in both sexual and asexual propagation [70]. Thus, *Hydra* can be considered immortal clonal animals. Every discrete polyp can propagate by asexual regeneration to form an unlimited number of offspring, similar to the propagation of an orchard from a single tree. Natural autotomy, when the head of the Hydrozoans separates from the foot and lives independently for days and releases sperm, while not mobile and autonomous as a medusa, it can passively perform and distribute its genetic material at the same time the foot regenerates a new head. Hydrozoans’ propagation is very similar to plants as both can reproduce asexually, and both can breed sexually when environmental conditions worsen. There is no evidence of what is the signal that sends Hydrozoan to propagate sexually. However, in the Hydrozoans, the stem cells used for tissue regeneration are also used to build the sexual gonads (testes and ovaries) every time the animal goes into a sexual propagation mode [71]. *Hydra oligactis* sexual individuals exhibited lower regeneration capacity compared to nonreproductives or asexuals animals [71].

## 4. Conclusions

Regeneration is not a mere curiosity nor an evolutionary relic from primitive multicellular organisms but a process necessary for the proper functioning of multicellular organisms. It is not just embryonic events that remain in various tissues of a few kinds of adult organisms.

All multicellular organisms possess a certain degree of tissue regeneration ranging from wound repair to whole-body renewal. However, sexual maturation causes a decline in regeneration capacity and rate in all multicellular organisms. Schaible et al. [72] suggest that asexual reproduction in *Hydra* allows them to escape aging, but the metabolic cost is high. The regenerative capacity can lead to an extended lifespan under asexual proliferation but requires elevated cell proliferation rates.

Plants regeneration process requires metabolic energy input, as exemplified by the fact that plant explants require sugar (sucrose or glucose) in the media and become a sugar sink during regeneration. The cellular competition between cell types somatic or germline idea is not mutually exclusive from the resource allocation theory. Both thoughts explain the observation that tissue regeneration and sexual reproduction are separate during plants’ and animals’ life cycles. The selective forces that work at the level of cells in plants were yet to be investigated. In animals, it is mostly theory due to the lack of a sound model system. It will indeed have to be well-backed by evidence to show that the cellular-level selective forces are more substantial than (or on par with, etc.) whole-body selective forces. I postulate that the ability to regenerate tissue or a whole body in multicellular organisms is thus maybe linked to the preferred mode of proliferation determined by environmental conditions of the organism or tissue. Once the conditions favoring sexual propagation shut down, clonal reproduction and regeneration can preside after diverting maintenance costs from sexual propagation to regeneration and vice versa. As I work on plants, we are now building a models system in tobacco plants to test the allocation of resources during regeneration and sexual reproduction. These tobacco plants will have visual markers (GFP, vital dyes, etc.) to visualize the movement of sugar and other metabolites during processes such as rooting (regeneration) and reproductive flowering.

A distinct negative correlation is observed all over the animal kingdom between regenerative ability, particularly whole body regeneration, and obligate sexuality [73]. Fields and Levin [73] suggest that stemness of germlines in multicellular organisms (in their case, animals only) is the result of gametic or somatic germlines actively repressing totipotent germlines, thus inhibiting or reducing regeneration and ending with sexual propagation only [73]. How do gametic germlines actively repress totipotent germlines? If we consider that totipotent germline and non-germline stem-cells struggle in multicellular organisms and that struggle ended with the gametic germlines wining in the multicellular state, I argue based on the above evidence, that sex hormones in all multicellular organisms from metazoans to mammals and plants are the mode gametic germlines maintain repress totipotent germlines thus inhibiting or reducing regeneration.

While variation in regenerative properties from amputated salamander limb or severed planarian tail or shoot or root regeneration in plants are present in all organisms, the cells that perform the regeneration all have in common the ability to develop into the primary germline cells and to reproduce and neoform the original tissue that is lost. The dissimilarity between all organisms rests in how this pluripotency potential is regulated and limited in the adult organism. One primary regulator is the asexual or sexual stage of the organism, as manifested by the effect of sex hormones on regeneration capacity. Together with other sources of primary regulation such as age and environment, govern the total pluripotential of cells.

## Data Availability

All data are available within the manuscript.
